# Perceived barriers and enablers to the provision of diabetic retinopathy screening for young adults: a cross-sectional survey of healthcare professionals working in the UK National Diabetic Eye Screening Programme

**DOI:** 10.1136/bmjdrc-2021-002436

**Published:** 2021-11-05

**Authors:** Louise Prothero, Fabianna Lorencatto, Martin Cartwright, Jennifer M Burr, Philip Gardner, John Anderson, Justin Presseau, Noah Ivers, Jeremy M Grimshaw, John G Lawrenson, for the EROS Study Investigators, John Anderson

**Affiliations:** 1 School of Health Sciences, City, University of London, London, UK; 2 Centre for Behaviour Change, University College London, London, UK; 3 School of Medicine, University of St Andrews, St Andrews, UK; 4 Public Health England, London, UK; 5 Diabetes and Endocrinology, Homerton University Hospital, London, UK; 6 Barts and The London School of Medicine and Dentistry, Blizard Institute, London, UK; 7 Clinical Epidemiology Program, Ottawa Hospital Research Institute, Ottawa, Ontario, Canada; 8 School of Epidemiology and Public Health, University of Ottawa, Ottawa, Ontario, Canada; 9 Women’s College Research Institute, Toronto, Ontario, Canada; 10 Department of Family and Community Medicine, University of Toronto, Toronto, Ontario, Canada; 11 Department of Medicine, University of Ottawa, Ottawa, Ontario, Canada

**Keywords:** young adult, diabetic retinopathy, qualitative research

## Abstract

**Introduction:**

Diabetic retinopathy screening (DRS) attendance in young adults is consistently below recommended levels. The aim of this study was to conduct a survey of screening providers in the UK Diabetic Eye Screening Programme (DESP) to identify perceived barriers and enablers to DRS attendance in young adults and elicit views on the effectiveness of strategies to improve screening uptake in this population.

**Research design and methods:**

Members of the British Association of Retinal Screening (n=580) were invited to complete an anonymous online survey in July 2020 assessing agreement with 37 belief statements, informed by the Theoretical Domains Framework (TDF) of behavior change, describing potential barrier/enablers to delivering DRS for young adults and further survey items exploring effectiveness of strategies to improve uptake of DRS.

**Results:**

In total, 140 (24%) responses were received mostly from screener/graders (67.1%). There was a high level of agreement that the DESP had a role in improving attendance in young adults (96.4%) and that more could be done to improve attendance (90.0%). The most commonly reported barriers related to TDF domains *Social influences* and *Environmental context and resources* including lack of integration of DRS with other processes of diabetes care, which limited the ability to discuss diabetes self-management. Other barriers included access to screening services and difficulties with scheduling appointments. Less than half (46.4%) of respondents reported having a dedicated strategy to improve screening uptake in young adults. Strategies perceived to be effective included: screening within the community; prompts/reminders and integrating eye screening with other diabetes services.

**Conclusions:**

Screening providers were concerned about screening uptake in young adults, although many programs lacked a dedicated strategy to improve attendance. Problems associated with a lack of integration between DRS with other diabetes care processes were identified as a major barrier to providing holistic care to young adults and supporting diabetes self-management.

**Significance of this study**

**What is already known about this subject?**

- In the UK, the National Health Service Diabetic Eye Screening Programme provides screening for all persons with diabetes aged 12 and over.
- Those younger than 35 years have been identified as having longer time intervals to first screening event, lower uptake of annual screening and an increased likelihood of missing three successive screening appointments.

**What are the new findings?**

- Individual healthcare professionals working in screening programs report the main barriers to attendance by young adults as the lack of integration of retinopathy screening with other processes of diabetes care, challenges accessing screening services and difficulties with scheduling appointments.
- Less than half of screening providers indicated that their program had a dedicated strategy in place to improve uptake in young adults.
- Strategies perceived by providers to be most effective for improving diabetic retinopathy screening (DRS) uptake in this group include screening within the community, prompts/reminders and integrating eye screening with other diabetes services.

**Significance of this study**

**How might these results change the focus of research or clinical practice?**

- A more tailored approach is needed to better support young adults to attend screening. To be most effective, interventions to improve screening uptake need to be targeted at individual and organizational levels.
- The findings of the current study can be combined with ongoing qualitative work on identifying barriers and enablers to DRS from the perspectives of young adults living with diabetes to further develop a package of behavior change intervention strategies to encourage attendance in this population group.

## Introduction

Diabetic eye disease, which comprises diabetic retinopathy and maculopathy, is one of the leading causes of sight loss among working age adults in the UK^1^ and throughout the world.^2^ From a public health perspective, a significant proportion of diabetes-related vision loss can be prevented through a systems-level approach that includes targeted education, a well-implemented community-level or national diabetic retinopathy screening (DRS) program, with timely referral pathways for further investigation, closer monitoring or treatment.^3^ Population-wide screening programs have been established in Iceland, Ireland and the UK, with regional and local screening programs in other parts of Europe.^4 5^ In the UK, DRS is managed by the National Screening Committee. In England, the National Health Service (NHS) Diabetic Eye Screening Programme (DESP) provides annual screening for approximately 3.3 million eligible people with diabetes aged 12 years and over through 57 regional DESPs. Screening clinics operate from a variety of fixed venues, for example, hospitals, community health centers and optometry practices as well as mobile screening units. Equivalent national programs in Scotland, Wales and Northern Ireland operate according to similar service specifications.

In the UK screening programs, the majority of people are screened through the ‘Routine Digital Screening’ pathway that uses digital retinal photography. Digital images of the retina are taken by ‘screeners’ and are then assessed by accredited ‘graders’. If sight-threatening retinopathy is identified, the person is either monitored more closely in a digital surveillance clinic or referred to the hospital eye service. Although uptake of screening is generally high (eg, 82.6% for England in 2018/2019), this overall figure masks variable uptake between regional programs and suboptimal attendance in particular demographic groups (eg, adults aged <35 years, mixed ethnicity groups, lower socioeconomic status groups).^5–7^

A recent retrospective analysis of attendance in three large urban screening programs in England serving a population of over 300 000 people with diabetes found that uptake rates were lowest among those aged 18–34 years. The odds of attending screening in this group were significantly less than the reference group of participants aged over 60, after controlling for other demographic variables (age, sex, ethnicity and socioeconomic deprivation).^7^ The study also analyzed new vision impairment certifications (Certificate of Vision Impairment) caused by diabetic eye disease in England and Wales from 2009 to 2019. This analysis showed that annual incidence of new certifications for vision impairment in young adults (aged <35 years) failed to show the net decline that has occurred in other age groups over the 10-year reporting period. There is good evidence that the more diabetes eye screening appointments are missed, the greater the risk that the next attendance will reveal sight-threatening disease.^8^

Increasing attendance to DRS among this vulnerable population group is thus a priority. A prerequisite for identifying how best to increase attendance rates is first understanding the reasons why young adults do or do not attend DRS. A recent systematic review of 69 studies reported barriers and enablers to DRS from the perspective of people with diabetes and healthcare providers.^9^ Barriers to DRS included, but were not limited to inaccurate diabetic registers, confusion between routine eye care and DRS, competing priorities, forgetting, fear of the procedure and screening results, diabetes denial and burnout, and financial concerns. Enablers of DRS included social support from relatives and friends, recommendations by healthcare professionals, and community-level media coverage.^9^ Although these findings provide a useful starting point for designing strategies to increase DRS, the review also highlighted a number of gaps in the available evidence base regarding barriers and enablers to DRS. Only two studies explored barriers and enablers from the perspective of young adults^10 11^ indicating that this is an under-researched population group and very few studies explored barriers and enablers to DRS from the perspective of healthcare professionals (HCPs) and systems.

Attending, providing, and encouraging DRS are all forms of human behavior.^12^ Therefore, exploring influences on DRS may be facilitated by the application of behavior change theories. Theories summarize the wealth of evidence in the wider literature, providing explicit statements summarizing processes that are hypothesized to regulate behavior. These can be used to explain and predict behavior, as well as identify how best to change behavior.^13^ The Theoretical Domains Framework (TDF)^14 15^ integrates constructs from 33 behavior change theories into 14 domains representing the wide range of individual, sociocultural and environmental influences on behavior (table 1). While the TDF has been used to explore influences on patient behaviors, including in the context of diabetes self-management^16 17^ and DRS specifically,^10 18^ it has been predominantly applied in implementation research to explore factors driving current clinical practice behaviors and what it would take to implement change in practice.^19^ To our knowledge, the TDF has not yet been applied to explore influences on attendance at DRS from the perspective of HCPs.

**Table 1:** Domains from the theoretical domains framework with examples of corresponding survey items

Domain (definition*)	Example belief statements from the survey
Knowledge (An awareness of the existence of something)	‘The guidelines and recommendations around DRS for people with diabetes in the UK are clear’
Skills (An ability or proficiency acquired through practice)	‘There is sufficient training available about DRS for professionals working within the DESP’
Beliefs about capabilities (Acceptance of the truth, reality or validity about an ability, talent or facility that a person can put to constructive use)	‘It is easy to discuss DRS with young adults’
Beliefs about consequences (Acceptance of the truth, reality, or validity about outcomes of a behavior in a given situation)	‘Improving attendance in young adults will help reduce vision loss’
Optimism (The confidence that things will happen for the best or that desired goals will be attained)	‘There is more we can do to try and increase attendance in young adults’
Intentions (A conscious decision to perform a behavior or a resolve to act in a certain way)	‘My screening service has plans in place to try and encourage attendance among young adults’
Goals (Mental representations of outcomes or end states that an individual wants to achieve)	‘There are more pressing priorities for the DESP than increasing attendance in young adults’
Reinforcement (Increasing the probability of a response by arranging a dependent relationship, or contingency, between the response and a given stimulus)	‘I am encouraged to try to increase attendance in young adults’
Memory, attention, decision-making (The ability to retain information, focus selectively on aspects of the environment and choose between two or more alternatives)	‘The DESP has strategies in place to try and remind young adults to attend’
Emotions (A complex reaction pattern, involving experiential, behavioral, and physiological elements, by which the individual attempts to deal with a personally significant matter or event)	‘I worry about screening attendance in young adults’
Social professional role/identity (A coherent set of behaviors and displayed personal qualities of an individual in a social or work setting)	‘DESP staff should play more of a role in discussing screening results with patients’
Environmental context and resources (Any circumstance of a person’s situation or environment that discourages or encourages the development of skills and abilities, independence, social competence and adaptive behavior)	‘The DESP is well integrated with specialist diabetes services in hospitals’
Social influences (Those interpersonal processes that can cause individuals to change their thoughts, feelings, or behaviors)	‘Communication across healthcare providers involved in diabetes care is poor’
Behavioral regulation (Anything aimed at managing or changing objectively observed or measured actions)	‘I receive feedback on my practice around DRS’

*TDF domain definitions from Atkins *et al*.^15^

DESP, Diabetic Eye Screening Programme; DRS, diabetic retinopathy screening; TDF, Theoretical Domains Framework.

The primary aim of the current study was therefore to apply the TDF to conduct a national survey exploring the barriers and enablers to attendance for DRS from the perspective of a representative sample of HCPs working in the UK DESP, with a specific focus on factors influencing the provision of DRS for young adults aged 18–34 years. As part of ongoing healthcare quality improvement efforts, DRS services may have also implemented various strategies to try and improve uptake of DRS in young adults and other population groups. It is important to document what has been tried so far, in order to learn from what has worked well and also what has not. Therefore, a secondary aim of this study was to identify current strategies implemented by UK DESPs to try and increase DRS attendance in young adults.

## Methods

### Design

A cross-sectional web-based survey.

### Participants and sample size

Eligible participants included all members from the current membership database of the British Association of Retinal Screening (BARS), a professional organization for those providing retinal screening services for people with diabetes. BARS supports continuing professional education through regular conferences, meetings, training days and educational activities. The membership of BARS includes retinal screeners/graders as well as optometrists, ophthalmologists and diabetologists involved in the NHS DESP. The survey took place in July 2020. At the time of recruitment, there were 580 ‘active’ members registered on the BARS database (members were considered active if they had logged into the BARS website within the last 12 months). As this was a descriptive survey, we did not have a predefined target sample size in mind and aimed to maximize response rate from as many current BARS members as possible.

### Materials: questionnaire

The survey was developed through an iterative process. A first draft was developed by the research team, which includes behavioral scientists with experience of developing TDF surveys, and then sent to two members of the project Research Advisory Group (a clinical ophthalmologist with experience in DRS and a former chairman of BARS with experience as a screener-grader and DESP manager) who provided feedback. Once face validity had been established, the survey was uploaded and pretested prior to distribution to BARS members. The full survey is available in the online supplemental material. In brief, the survey was fully anonymous and divided into three sections:

10.1136/bmjdrc-2021-002436.supp1 Supplementary data

Section 1: Role in the screening program (five questions) including which screening program worked for; primary role; duration of employment and HCPs most often worked with.

Section 2: Perceived barriers and enablers to delivering DRS for young adults and supporting (or enabling) their attendance at appointments. This included 37 items structured around the 14 domains of the TDF,^14^ with at least one item per domain. Items represented belief statements, to which respondents were asked to rate their extent of agreement using a 5-point Likert scale (Strongly agree → Strongly disagree). Table 1 includes the 14 domains, their definition, and a sample survey belief statement developed to assess that domain. Some of the generation of these belief statements was in part informed by the aforementioned systematic review on barriers and enablers to DRS.^9^

Section 3: Included seven items to assess which interventions or strategies have previously been used to try to improve young adults’ uptake of DRS. Respondents were shown a list of potential strategies (eg, mobile screening clinics, reminder letters, peer support groups), identified in part based on a previous systematic review of interventions to increase DRS^7^ and were asked to tick all that have been tried previously by their screening program. For those strategies that had been tried, participants were asked to rate on a 5-point Likert scale (Not effective → Extremely effective) how effective this strategy was in improving uptake of DRS among young adults. Lastly, this section also contained free text boxes where participants could list any other strategies that their service had trialed, or suggestions as to what else they felt could be done to improve the delivery of DRS to encourage attendance in young adults.

### Procedure

The survey was hosted online using Qualtrics survey software (https://www.qualtrics.com/uk/core-xm/survey-software/). Pretesting for technical quality and appearance was performed by members of the research team prior to sending a personalized explanatory invitation email to members of BARS, which contained a hyperlink to the survey. The survey was open for 4 weeks during which two follow-up reminders were sent. The survey was fully anonymous and participants consented to participate in the survey by completing a brief consent form on the survey home page. No incentives were offered to participate.

### Analysis

After the closure of the survey, all data were imported into an Excel spreadsheet. Respondents who either fully or partially completed section 1 describing their role within the screening program, but exited the survey before completing sections 2 and 3, were excluded from the analysis. Responses to the 5-point Likert scale in section 2 measuring agreement or disagreement with a series of statements on what influences the delivery of DRS were converted to a numerical score (Strongly agree=1; Somewhat agree=2; Neither agree or disagree=3; Somewhat disagree=4; Strongly disagree=5) to calculate the mean and SD. As the survey included a mixture of positively and negatively worded belief statements, negatively worded items (see table 2) were reverse scored so that for all items, lower scores indicated the perception that the belief statement represented an enabler to DRS, while higher scores indicated the perception that the belief statement represented a barrier to DRS. Data were summarized using descriptive statistics (ie, percentages (n), or mean and (SD) as appropriate). An overall score for each of the 14 TDF domains was calculated by averaging score across items mapping to that domain.^20^

**Table 2:** Means (SDs) and percentage agreement for each belief statement

Domain	Mean	SD	n	Agreement (%)
‘Thinking about your role in providing and/or supporting diabetic retinopathy screening for young adults with diabetes (aged 18–34 years), please rate your agreement with the following statements:’				
Knowledge				
‘The guidelines and recommendations around DRS for people with diabetes in the UK are clear’	2.23	1.02	139	66.9
‘The standards around DRS for people with diabetes in the UK are clear’	2.07	1.09	139	71.2
‘I am aware of attendance patterns in young adults in my DESP'	1.87	0.82	139	82.0
‘I am aware of a patient’s current diabetes self-management (ie, Hba1c)’	3.23	1.31	140	34.3
‘It would be helpful to know how patients are currently managing their diabetes’*	4.30	0.81	139	85.6
Skills				
‘There is sufficient education available about DRS for professionals working within the DESP’	2.18	1.10	140	71.4
‘There is sufficient training available about DRS for professionals working within the DESP’	2.06	1.10	139	74.8
Social/professional role and identity				
‘The DESP has a role to play in encouraging attendance among young adults’	1.40	0.63	140	96.4
‘The roles and responsibilities of different healthcare professionals involved in caring for people with diabetes is clear’	2.65	1.05	140	51.4
‘It is the responsibility of other healthcare professionals to encourage attendance in young adults with diabetes'*	3.50	1.11	140	53.6
‘DESP staff should play more of a role in discussing screening results with patients’	2.14	1.10	140	70.0
‘I would like the ability to refer patients to additional support for their diabetes’*	4.50	0.67	139	91.4
Optimism				
‘There is more we can do to try and increase attendance in young adults’	1.54	0.71	140	90.0
Beliefs about capabilities				
‘It is easy to discuss DRS with young adults’	2.69	1.12	137	46.0
Beliefs about consequences				
‘Improving attendance in young adults will help reduce vision loss’	1.08	0.30	140	99.3
Reinforcement				
‘I am encouraged to try to increase attendance in young adults’	2.24	1.04	140	63.6
Intention				
‘My screening service has plans in place to try and encourage attendance among young adults’	2.47	1.07	140	52.9
Goals				
‘Supporting attendance in young adults is a priority for the DESP’	2.13	0.90	140	66.4
‘There are more pressing priorities for the DESP than increasing attendance in young adults’*	2.58	0.95	139	17.3
‘My screening service has targets around screening attendance’	1.21	0.49	140	96.4
Memory, attention and decision processes				
‘The DESP has strategies in place to try and remind young adults to attend’	2.31	0.95	140	70.0
Environmental context and resources				
‘The DESP is well integrated with ophthalmology services’	2.15	1.21	140	71.4
‘The DESP is well integrated with specialist diabetes services in hospitals’	2.86	1.17	140	40.0
‘The DESP is well integrated with GP practices in primary care’	2.44	1.01	140	61.4
‘Problems with re-scheduling appointments impacts young adults’ attendance’*	3.76	1.02	140	65.7
‘The DESP has sufficient staff to provide DRS to patients’	2.66	1.29	139	55.4
‘The DESP have sufficient time to provide DRS to patients’	2.35	1.25	140	67.1
‘The DESP have sufficient resources to provide DRS to patients’	2.55	1.30	140	61.4
‘Incomplete or inaccurate registers make it more difficult for the DESP to support DRS in young adults’*	4.11	1.04	140	77.1
‘Transient populations make it more difficult for the DESP to support DRS in young adults’*	3.83	0.85	140	63.6
‘Accessibility of the screening service impacts young adults’ attendance’*	3.89	1.08	139	72.1
‘DRS appointments are a good opportunity to discuss diabetes management with patients’	2.41	1.35	140	58.6
Social influences				
‘Communication across healthcare providers involved in diabetes care is poor’*	3.72	0.95	140	62.9
‘Language is a barrier to supporting DRS’*	3.44	1.04	140	52.9
Emotion				
‘I worry about screening attendance in young adults’	1.67	0.76	140	85.0
Behavioural regulation				
‘I receive feedback on my practice around DRS’	2.21	1.04	138	67.4
‘My colleagues and I discuss screening attendance and how to improve it’	2.02	1.02	140	74.3

The mean scores correspond to the extent to which participants agreed with each statement using a 5-point Likert scale (strongly agree=1; somewhat agree=2; neither agree nor disagree=3; somewhat disagree=4; strongly disagree=5), after scores have been reversed where applicable.

*Belief statements in italics have been reverse scored. The level of agreement is based on those strongly or somewhat agreeing with the belief statement.

DESP, Diabetic Eye Screening Programme; DRS, diabetic retinopathy screening; GP, General Practitioner (Family Physician).

Free text responses regarding suggestions for intervention strategies to encourage screening were analyzed qualitatively. Suggested interventions were deductively coded using the behavior change wheel (BCW) as a coding framework.^21^ The BCW is a synthesis of 19 frameworks of different types of behavior change interventions. It specifies nine broad intervention functions (education, training, modeling, persuasion, environmental restructuring, incentivization, coercion, restriction, enablement) and seven policy options (eg, mass media, legislation, service provision). Suggested interventions were deductively coded into the intervention functions and policy options specified in the BCW by one author (JGL) and then checked by a second author with expertise in the application of the BCW (FL).

### Mapping barriers to behavior change techniques

TDF domains representing perceived barriers and enablers reported by HCPs to delivering DRS were subsequently mapped to broad intervention functions in the BCW and more specific behavior change techniques (BCTs)^22^ to identify candidate intervention strategies to improve DRS uptake. This was done using a stepwise process by consulting the following tools and sources of evidence:

1. Published matrices that pair TDF domains with broad intervention functions in the BCW to suggest which intervention types are appropriate to address barriers and enablers within each TDF domain.^21^
2. The Theory and Techniques Tool^23^ which uses expert consensus and evidence in the literature to pair more granular BCTs (eg, goal setting, problem solving, feedback on behavior) from a 93-item taxonomy^22^ with TDF domains.

BCTs that have previously been shown to be effective for targeting HCP behavior to promote DRS in the general population of people with diabetes were identified using evidence from an existing Cochrane systematic review.^24^

## Results

### Response rate

In total, 154 respondents completed all or part of section 1. Fourteen participants did not progress to complete sections 2 and 3. The analysis is therefore based on 140 respondents who completed all three sections of the survey (this corresponds to a response rate of 24.1%). Rate of missing data ranged from 0% to 25.7% for each questionnaire item (mean 2.3%).

#### Section 1: respondent characteristics

Amongst those providing an affiliation, 77 respondents were affiliated to 40 of the 57 DESPs in England, 6 respondents were from Diabetic Eye Screening (DES) Wales, 11 from the Northern Ireland DESP and 1 from the national screening program in Scotland. In terms of their primary role, the largest proportion of responders (67.1%) were either screeners (12.1%, n=17) (taking photographs of the retina), graders (3.6%, n=5) (grading images) or screeners-graders (taking photographs and grading images) (52.1%, n=73). 9.3% (n=13) were optometrists or ophthalmologists with a variety of clinical roles in the DESP (eg, clinical leads, grading using slit lamp biomicroscopy), 10% (n=14) were involved in operational management in the DESP, 8.6% (n=12) were responsible for quality assurance or fail-safe and 4.2% (n=6) were working in administration or stakeholder engagement. 61.4% (n=86) of respondents had been working in DRS for 5 years or more.

#### Section 2: perceived barriers and enablers to delivering DRS

The descriptive statistics pertaining to the level of agreement with each belief statement, and the calculated domain score, are summarized in table 2 and online supplemental table S1.

The domains with the highest domain scores and thus representing barriers to DRS were *Social influences* (mean=3.58, SD=1.00) and *Environmental context and resources* (mean=3.00, SD=1.36) (see online supplemental table for mean scores for all domains). However, overall, most of the domains represented mixed influences on delivery of DRS to young adults—including both reported barriers and enablers. For instance, within the domain *Knowledge*, there were several enablers—with many respondents indicating they were both familiar with guidelines and recommendations for screening (66.9% strongly or somewhat agreeing with this belief statement), and 82% were aware of patterns of attendance of young adults within their service (82%). However, there were also barriers within *Knowledge—*with only a third of respondents indicating that they knew how well young adults under their care were currently managing their diabetes (most respondents agreed having this information would be helpful).

Enablers to delivering DRS included an almost universal agreement that the DESP has a role in improving screening attendance in young adults (96.4%) (TDF domain: *Social professional role/identity)* and the belief that improved attendance would help reduce vision loss (99.3%) (*Beliefs about consequences*). Screening attendance in young adults was viewed as a concern (85.0%) (*Emotions*) and respondents felt that more could be done to improve uptake in this population (90.0%) (*Optimism*), with two-thirds agreeing that this should be a priority for the DESP (66.4%) (*Goals*). Approximately half of respondents agreed that their screening program had plans in place to encourage attendance in young adults (52.9%) (*Intentions*) and approximately two-thirds agreed that they were individually encouraged to increase attendance rates (63.6%) (*Reinforcement*). Most participants indicated that sufficient education and training was available for HCPs on DRS (71.4% and 74.8% agreement, respectively).

However, numerous barriers to providing DRS were also highlighted. Less than half agreed with the statement ‘It is easy to discuss diabetic retinopathy screening with young adults’ (46.0%) (*Beliefs about capabilities*). Most respondents (63%) felt that the transient nature of young adults, who might be frequently moving between accommodation due to studies or employment, made it difficult to provide DRS to this population group (*Environmental context and resources*). Numerous service provision-level barriers were flagged, mostly within the domain *Environmental context and resources*. These included having insufficient staff to deliver DRS (55.4%), problems related to the accessibility of the screening service (72.1%), and problems with rescheduling appointments (65.7%). Although there was a general feeling that screening programs were well integrated with hospital ophthalmology, participants reported a lack of integration with other areas of diabetes care, particularly specialist diabetes services (40%). Communication across healthcare providers involved in diabetes care was seen as poor by almost two-thirds of participants (62.9%) (*Social influences*). Participants also felt limited by their role, with over 90% of respondents indicating that they would like the ability to refer people to additional support for their diabetes and 70% indicating that ‘DESP staff should play more of a role in discussing screening results with patients’ (*Social professional role/identity*).

#### Section 3: interventions/strategies in place to improve DRS attendance

Less than half of the respondents (46.4%, n=65) indicated that their screening program had a specific strategy in place to improve screening uptake in young adults. The main reasons given by respondents for not having a strategy were ‘lack of time and resources’.

Table 3 summarizes the strategies respondents indicated their DESP targeted at young adults with diabetes to try and increase uptake of DRS, and the extent to which these were perceived to be effective.

**Table 3:** Strategies targeted at young adults with diabetes to try and improve DRS uptake and perceptions of their effectiveness

Strategy directed at person with diabetes	Adopting strategy	Perceived effectiveness					
	n (%)	Extremely effective	Very effective	Moderately effective	Slightly effective	Not effective	Mean (SD)*
Dedicated clinics for young people	35 (34.3)	4 (11.4)	11 (31.4)	11 (31.4)	6 (17.1)	3 (8.6)	3.20 (1.13)
Mobile screening units	57 (55.9)	9 (15.7)	9 (15.7)	26 (45.6)	12 (21.1)	1 (1.8)	3.23 (1.02)
Screening within the community	101 (99.0)	19 (18.8)	33 (32.7)	40 (39.6)	9 (8.9)	0 (0.0)	3.61 (0.89)
Integrating eye screening with other diabetes services (eg, ‘one-stop shop’ clinics)	46 (45.1)	17 (37.0)	13 (28.3)	13 (28.3)	2 (4.3)	1 (2.2)	3.94 (1.02)
Self-management programs/training for people with diabetes	47 (46.1)	3 (6.4)	19 (40.4)	17 (36.2)	6 (12.8)	2 (4.3)	3.36 (0.85)
Provision of information about diabetic retinopathy	99 (97.1)	10 (10.1)	20 (20.1)	42 (42.4)	25 (25.3)	2 (2.0)	3.11 (0.97)
Peer support groups	44 (43.1)	3 (6.8)	15 (34.1)	18 (40.9)	8 (18.2)	0 (0.0)	3.30 (0.85)
Prompts/reminders (eg, text messages, letters, phone calls)	102 (100)	27 (26.5)	35 (34.3)	28 (27.5)	11 (10.8)	1 (1.0)	3.75 (1.00)
Continuing to offer screening appointments to people who do not attend	102 (100)	16 (15.7)	17 (16.7)	40 (39.2)	22 (21.6)	7 (6.9)	3.13 (1.13)

*Mean score represents effectiveness of strategy on a 5-point scale (extremely effective=5; not effective=1).

DRS, diabetic retinopathy screening.

The most commonly used interventions targeting young adults with diabetes were the provision of information about diabetic retinopathy (97.1%), prompts or reminders to attend the appointment (100%) as well as continuing to offer screening appointments to people who do not attend (100%). Three strategies were endorsed as ‘very effective’ or ‘extremely effective’ by more than 50% of respondents: screening within the community (51.5%), prompts/reminders (eg, text messages, letters, phone calls) (60.8%), and integrating eye screening with other diabetes services (eg, ‘one-stop shop’ clinics) (65.3%). The strategies with the lowest perceived effectiveness were provision of information about diabetic retinopathy (30.2%), mobile screening units (31.4%) and continuing to offer screening appointments to people who do not attend (32.4%).

Approximately 20% of respondents (n=30) provided information on strategies to encourage screening attendance that were not in the provided list including offering appointments in the evenings and weekends, contacting the person with diabetes directly by phone, and providing information on screening to schools, colleges and universities.

Table 4 summarizes strategies targeted at HCPs to try and support delivery of DRS to young adults, and their perceived effectiveness. The most commonly adopted strategies operating at the level of the HCP or screening program were the use of electronic patient registers (95%), audit and feedback (92%) and clinical education (85%). Three strategies were perceived as extremely or very effective by more than 50% of respondents: clinical education (58.4%), the use of electronic registers (56.5%) and telemedicine (eg, EyePACS)/virtual clinics (51.6%). Audit and feedback was perceived as extremely or very effective by approximately half of participants (47.5%).

**Table 4:** HCP-targeted strategies used to improve screening uptake in young adults and perception of their effectiveness

Healthcare Professional (HCP) strategy	Adopting strategy	Perceived effectiveness					
	n (%)	Extremely effective	Very effective	Moderately effective	Slightly effective	Not effective	Mean (SD)
Clinical education	89 (87.3)	16 (18.0)	36 (40.4)	27 (30.3)	10 (11.2)	0 (0.0)	3.65 (0.91)
Audit and performance feedback (eg, feedback on number of patients screened per month)	97 (95.1)	18 (18.6)	28 (28.9)	40 (41.2)	11 (11.3)	0 (0.0)	3.55 (0.92)
Electronic registers (which hold information about patients and their eye screening appointments)	99 (97.1)	24 (24.2)	32 (32.3)	33 (33.3)	9 (9.1)	1 (1.0)	3.70 (0.97)
Telemedicine (eg, EyePACS)/virtual clinics	31 (30.4)	4 (12.9)	12 (38.7)	14 (45.2)	1 (3.2)	0 (0.0)	3.61 (0.76)

Table 5 summarizes participants’ suggestions for additional strategies that could be used to encourage DRS uptake in young adults, coded according to intervention functions and policy options from the BCW.^21^ Free text responses were received from 102 (72.9%) of respondents. The majority of suggestions were primarily targeting young adults with diabetes rather than HCPs, such as strategies that aimed to increase knowledge of diabetic retinopathy and the importance of screening (*BCW intervention function: Education*), active encouragement from General Practitioners (GPs)/diabetes nurses, promoting self-management (*BCW intervention function: Persuasion*), improved communication—particularly the use of social media, targeted campaigns to increase awareness (*BCW policy category:* C*ommunication/marketing),* or changes in the way that the service is delivered through the selective use of dilating eye drops in young adults, allowing more flexibility in terms of evening or weekend appointments and self-booking of appointments (*BCW intervention function*: *Environmental restructuring*; *BCW policy category: Service provision*).

**Table 5:** Respondent suggestions as to how screening uptake in young adults could be improved. Interventions were coded to the intervention and policy taxonomy used in the behavior change wheel

What else do you think could be done to encourage attendance in young adults? (n=102)		
	Frequency n (%)	Examples
**Intervention**		
Education	23 (22.6)	‘More education about the long terms risks, and the asymptomatic nature of Diabetic retinopathy’ ‘More education for General Practioners (GPs)’
Persuasion	0 (0.0)	N/A
Incentivization	3 (2.9)	‘Re-imbursement of travel costs as pts can't drive themselves with dilation’
Coercion	0 (0.0)	N/A
Training	1 (0.98)	‘More training’
Restriction	0 (0.0)	N/A
Environmental restructuring	1 (0.98)	*‘*Ensuring they are aware that a drop-in appointment is possible’ ‘More freedom to discuss consequence of non-attendance with patients in clinic’
Modeling	0 (0.0)	N/A
Enablement	11 (10.8)	‘Active encouragement from GPs/Diabetic nurses’ ‘Chasing up young adults who have not attended to get them rebooked and see if there is anything the programme can do to help’
**Policy**		
Communication/marketing	28 (27.5)	‘Social media campaigns aimed specifically at young people - celebrity endorsement of DRS’
Guidelines	0 (0.0)	N/A
Fiscal	0 (0.0)	N/A
Regulation	2 (1.96)	‘Running audits and reports into young patients who have not attended’
Legislation	0 (0.0)	N/A
Environmental/social planning	0 (0.0)	N/A
Service provision	58 (56.9)	*‘*A joint up service. All diabetic services working together’ ‘A mobile clinic, weekend appointments as young adults work/childcare during the week so evening clinics not enough’

DRS, diabetic retinopathy screening; N/A, not applicable.

In terms of suggestions that were targeted at HCPs, the majority of suggestions related to improved communication between the screening program and members of the primary care and diabetes teams and increasing HCP knowledge and understanding of diabetic eye disease.

### Mapping to candidate BCTs

The results from the TDF to BCW and BCT mapping exercise with examples of how candidate intervention strategies could be operationalized are presented in online supplemental table S2. Examples of potentially relevant intervention functions from the BCW included persuasion, education and environmental restructuring. Ten BCTs were identified that could potentially be used in intervention design; however, only three of these have been previously used in trials of interventions targeted at HCPs to increase DRS in general populations of people living with diabetes.^24^ Interventions varied in complexity and were targeted at young adults with diabetes and HCPs. In some cases, delivery of the intervention required a change in the organization of the screening program or diabetes care pathway.

## Discussion

This study is the first in-depth exploration of the barriers and enablers to uptake of DRS screening in young adults from the perspective of HCPs working in the UK NHS DESP. To identify barriers and enablers we used behavioral science frameworks including the TDF and BCW.^14 21^ The most commonly reported barrier domains were *Environmental context and resources* and *Social influences*. This is consistent with findings from the broader literature.^9 10 18^ In the UK, although DRS is one of the nine processes of diabetes care recommended by the National Institute for Health and Care Excellence,^25 26^ eye screening is administered as one of the NHS population screening programs and consequently is not fully integrated with the other aspects of diabetes care. Furthermore, DRS is no longer one of the diabetes indicators in the quality and outcomes framework of the General Medical Services contract. This arrangement explains the finding in the present study that although participants felt that their screening program was integrated with ophthalmology services, it was less well integrated with GP practices and with specialist diabetes services in hospitals. Furthermore, there was a perceived lack of clarity among staff working within the DESP on the roles and responsibilities of different HCPs involved in diabetes care and a lack of communication between the DESP and other diabetes healthcare providers.

A number of barriers were identified relating to young adults’ access to screening services and difficulties with scheduling screening appointments. Similar barriers were reported in a recent systematic review of barriers and enablers to DRS,^9^ including competing time demands with work commitments and family responsibilities, compounded by a lack of appointment flexibility. A high percentage of responders to the survey agreed that transient populations and incomplete or inaccurate registers also impacted on the screening programs’ ability to support DRS in young adults. A recent study of repeat non-attenders to the Welsh national screening program found that frequent house moves were associated with a failure to attend three consecutive annual screening appointments.^27^ Residential moves between different geographic areas are more likely in the young adult population as they move for study or employment, which could be a contributing factor to suboptimal DRS attendance in this population. Although participants reported that they were limited in their ability to discuss diabetes self-management due to a lack of information on how attendees are managing their diabetes, approximately 90% of respondents indicated that they would like the ability to refer onwards for further support.

In terms of enablers to DRS, there were high levels of agreement that screening attendance in young adults was a concern, should be a priority for the DESP and there was a general view that screening uptake in this population could be improved. DESP staff also reported that they would like to be more involved in discussing screening results with young adults. The finding that DESP staff are engaged and highly motivated to try and improve the delivery and uptake of DRS in young adults and expand their role provides an opportunity to build on these enablers to develop and test interventions to improve screening attendance. A number of BCTs have been shown to be effective in improving attendance for DRS in randomized controlled trials^24^ and there is a high degree of theoretical coherence between the components of these interventions and the theoretical determinants of screening attendance.^28^

Less than half of those responding to the survey indicated that their screening program had a dedicated strategy in place to improve uptake in young adults. One of the strategies that was viewed as being most effective was ‘one-stop shop’ clinics that integrated eye screening with other diabetes care processes. This aligns with the identified barriers to screening attendance, including the lack of integration with other services and poor communication across service providers in other aspects of diabetes care. Integrating diabetes care involves delivering care and support coordinated around the needs of the person with diabetes, ensuring that all parts of the system work together to deliver all components of the care pathway. DRS is one of many competing demands experienced by a person with diabetes. Sustained engagement with self-management behaviors is a critical element in achieving glycemic control and minimizing risk of complications,^16^ and therefore despite the challenges, integrating DRS with other aspects of diabetes care makes sense in terms of making these behavioral processes easier for people with diabetes. There is currently no high-quality evidence that one-stop clinics improve DRS uptake specifically in young adults; however, ‘collaborative case management’, which coordinates processes of diabetes care, has been shown to improve uptake in trials of a general population of adults with diabetes.^24^

Significantly, a number of the most commonly used strategies, for example, provision of information about DRS and continuing to offer appointments to people who do not attend, were perceived as being less effective compared with other strategies (ie, screening within the community, one-stop shops, self-management training for people with diabetes, prompts/reminders (eg, text messages, letters, phone calls)). Offering further appointments to non-attenders assumes that the contact information on the register is correct, which links to the identified barrier of accuracy of screening registers.

The majority of the most commonly used HCP-targeted strategies to improve attendance were rated as either ‘extremely effective’ or ‘very effective’, with clinical education and the use of electronic registers reported to be most effective. This is consistent with previous studies of interventions to improve DRS attendance.^24 28^

### Strengths and limitations of the study

The strength of the present study is its wide coverage, with responses received from approximately 70% of the DESPs operating in England, together with responses from the national screening programs in Wales, Scotland and Northern Ireland. Furthermore, the survey was able to gather the views of a wide range of professionals working within UK screening programs, including screeners, graders, optometrists, ophthalmologists as well as those involved in the operational management of the DESP. Within each of the four nations local screening programs deliver DRS to a common service specification, there is a reasonable amount of autonomy to allow local programs to develop their own strategies to increase screening uptake. Structuring the survey items on barriers and enablers around the 14 domains of TDF helped ensure that the broad range of potential individual, sociocultural and environmental influences on DRS provision was considered. The current study has collected data on a number of strategies currently used to improve DRS uptake in young adults and views on the relative effectiveness of these strategies. Classifying these using the BCW helped identify alignment of strategies with the barriers/enablers identified within TDF domains. These frameworks have recently been applied to design an intervention to improve self-management in young adults with type 2 diabetes^28 29^ and to increase the uptake of DRS in Ireland.^12^

A limitation of the study is the relatively low response rate (24%). However, the breakdown of respondents into their respective professional groups is consistent with the membership database of BARS, suggesting that there were no substantial differences in response rates between professional groups. However, we cannot exclude the possibility of non-response bias.

## Conclusions

The National DESPs in the UK have been very successful in ensuring that the vast majority of the eligible population of people with diabetes receive retinopathy screening in a timely manner.^4^ In 2014, a study of the causes of blindness in England revealed that for the first time in five decades, diabetic eye disease was no longer the most common cause of blindness in the working age population.^29 30^ However, there is considerable variation in screening uptake among age groups. People with diabetes younger than 35 years have been identified as having longer time intervals from registration with the screening program to first screening event,^5 7^ lower uptake of annual screening^6 7^ and an increased likelihood of missing three successive screening appointments.^7 27^ This suggests that a more tailored approach is needed to better support young adults to attend screening. To be most effective, behavior change interventions to improve screening uptake will need to be targeted at individual and organizational levels^30 31^ and are likely to vary in scope and intensity. Examples of how BCTs relating to barriers identified in the current study could be operationalized can be found in online supplemental table S2. The findings of the current study will be combined with ongoing qualitative work on identifying barriers and enablers to DRS from the perspectives of young adults living with diabetes to further develop a package of behavior change intervention strategies to encourage attendance in this population group.

## Data Availability

Data are available upon reasonable request. All data relevant to the study are included in the article or uploaded as supplementary information.
