# Supplementary material for: Perceived barriers and enablers to the provision of diabetic retinopathy screening for young adults: a cross-sectional survey of healthcare professionals working in the UK National Diabetic Eye Screening Programme
Source: BMJ Open Diabetes Res Care. 2021 Nov 5;9(2):e002436. doi: 10.1136/bmjdrc-2021-002436 (PMC8573632; doi:10.1136/bmjdrc-2021-002436)
Supplement: Supplementary data [file bmjdrc-2021-002436supp001.pdf]

Online Supplementary Material

Copy of BARS survey

How can the provision of diabetic retinopathy screening for young adults be improved?

This survey is part of a wider study (the EROS Study) which aims to identify barriers and enablers to diabetic retinopathy screening (DRS) in young adults (aged 18-34 years) with Type 1 and Type 2 Diabetes. We are exploring factors from the perspective of people with diabetes as well as healthcare professionals involved in providing screening. **The purpose of the survey is to explore your views about factors influencing the provision of DRS to young adults, and what can be done to encourage attendance.** The information you provide will help us to generate recommendations to the screening programme about potential interventions to encourage young adults' attendance.

The survey is divided into three parts: 'Section 1' asking about your role within the NHS Diabetic Eye Screening Programme (DESP), 'Section 2' exploring your views about what might influence the delivery of screening, and 'Section 3' asking about procedures in place to support attendance within your DESP. **The survey will take approximately 15 minutes to complete.** There are no right or wrong answers. You can select 'Not applicable' or leave blank any questions you do not wish to answer. If you wish to take part, you will be taken to the next screen and asked to fill in a brief consent form. **The information you provide is anonymous and fully confidential.** No one from your place of employment will be aware of your decision to complete the survey or responses.
You can withdraw at any time before, during or after the survey, without giving a reason.

This study has been approved by Wales Research Ethics Committee 2 (REC reference: 19/WA/0228). If you have any questions or concerns regarding completing the survey, please e-mail the study researcher: Louise.Prothero.3@city.ac.uk
Thank you for taking the time to take part in our survey,

The EROS Study team

[Consent statements]

Section 1: Your role

1. Which DESP do you work for? *[drop down list of DESPs]*
2. What is your role within the DESP? (please tick all that apply) *[drop down list of role titles: retinal screeners, graders, administrative staff, failsafe staff, programme managers, optometrists, ophthalmologists, other please specify]*

**Free text box**

3. How many years have you worked in this role? *[drop down list: < 1 year; 1-<5 years, 5-<10 years, 10 years +]*
4. What is your role in the provision of DRS for people with diabetes? (please tick all that apply) *[drop down list of options: taking photographs of the retina, grading images, , treating diabetic retinopathy, providing failsafe, administrative responsibilities, responsibility for the operational running of the DESP, clinical responsibility for the DESP, other please specify]*

**Free text box**

5. What types of other healthcare professionals do you most often work with in relation to DRS? (please tick all that apply) *[drop down list of different HCPs across care settings: General Practitioners, Diabetes Nurses, ,Ophthalmologist, Optometrist, Diabetologists, Psychologists, Transition Team, Stakeholder Engagement Managers, other please specify]*

**Free text box**

Section 2: Your views about what influences the delivery of DRS

Thinking about your role in providing and/or supporting diabetic retinopathy screening for young adults with diabetes (aged 18-34 years), please rate your agreement with the following statements:

5-point Likert scale (Strongly disagree, Somewhat agree, Neither agree or Disagree, Somewhat disagree, Strongly disagree).

**Theoretical domain**	**Item**
1. Knowledge	'The guidelines and recommendations around DRS for people with diabetes in the UK are clear'<br><br>'The standards around DRS for people with diabetes in the UK are clear'<br><br>'I am aware of attendance patterns in young adults in my DESP'<br><br>'I am aware of patients' current diabetes self-management (i.e. Hba1c)'<br><br>'It would be helpful to know how patients are currently managing their diabetes'
2. Skills	'There is sufficient education available about DRS for professionals working within the DESP'<br><br>'There is sufficient training available about DRS for professionals working within the DESP'
3. Social/professional role and identity	'The DESP has a role to play in encouraging attendance among young adults'<br><br>'The roles and responsibilities of different healthcare

	professionals involved in caring for people with diabetes is clear’<br>‘It is the responsibility of other healthcare professionals to encourage attendance in young adults with diabetes’<br>‘DESP staff should play more of a role in discussing screening results with patients’<br>‘I would like the ability to refer patients to additional support for their diabetes’
4. Optimism	‘There is more we can do to try and increase attendance in young adults’
5. Beliefs about capabilities	‘It is easy to discuss DRS with young adults’
6. Beliefs about consequences	‘Improving attendance in young adults will help reduce vision loss’
7. Reinforcement	‘I am encouraged to try to increase attendance in young adults’
8. Intention	‘My screening service has plans in place to try and encourage attendance among young adults’
9. Goals	‘Supporting attendance in young adults is a priority for the DESP’<br>‘There are more pressing priorities for the DESP than increasing attendance in young adults<br>‘My screening service has targets around screening attendance’
10. Memory, attention and decision	‘The DESP has strategies in place to try and remind young adults to attend’

processes	
11. Environmental context and resources	'The DESP is well integrated with ophthalmology services'<br><br>'The DESP is well integrated with specialist diabetes services in hospitals'<br><br>'The DESP is well integrated with GP practices in primary care'<br><br>'Problems with re-scheduling appointments impacts young adults' attendance'<br><br>'The DESP has sufficient staff to provide DRS to patients'<br><br>'The DESP have sufficient time to provide DRS to patients'<br><br>'The DESP have sufficient resources to provide DRS to patients'<br><br>'Incomplete or inaccurate registers make it more difficult for the DESP to support DRS in young adults'<br><br>'Transient populations make it more difficult for the DESP to support DRS in young adults'<br><br>'Accessibility of the screening service impacts young adults' attendance'<br><br>'DRS appointments are a good opportunity to discuss diabetes management with patients'

12. Social influences	'Communication across healthcare providers involved in diabetes care is poor'<br><br>'Language is a barrier to supporting DRS'
13. Emotion	'I worry about screening attendance in young adults'
14. Behavioural regulation	'I receive feedback on my practice around DRS'<br><br>'My colleagues and I discuss screening attendance and how to improve it'

Section 3: Interventions/strategies in place to improve diabetic eye screening attendance

We wish to learn more about what interventions or strategies DESPs have put into place to try and improve attendance among young adults (age 18-34 years).

1. Within your programme, are there any strategies in place to improve young adults' uptake of diabetic eye screening?

Yes	No	Unsure

2. Please indicate why you think no strategies have been put in place (please tick all that apply) *[drop down list of reason: lack of resources, lack of time, not a priority, attendance is already high, lack of support from colleagues/managers, other please specify]*

**Free text box**

3. Please indicate any strategies in place to improve young adults' uptake of diabetic eye screening (please tick all that apply)

[Drop down list of strategies targeted at people with diabetes]:

Dedicated clinics for young people

Mobile screening units

Screening within the community

Integrating eye screening with other diabetes services (e.g. 'one-stop shop' clinics)

Educational programmes/training for people with diabetes to increase awareness of diabetic retinopathy and promote self-management

Provision of information about diabetic retinopathy

Peer support groups

Continuing to offer screening appointments to people who do not attend

Prompts/reminders (e.g. text messages, letters, phone calls)

No strategies in place

Other (please specify below)

[Drop down list of strategies targeted at professionals working within the DESP]

Clinical education

Audit and performance feedback (e.g. feedback on number of patients screened per month)

Electronic registers (which hold information about patients and their eye screening appointments)

Telemedicine (e.g. EyePACS)/Virtual clinics

No strategies in place

Other (please specify below)

If you selected 'Other' please provide a description of any strategies in place to improve young adults' uptake of diabetic eye screening in the box below

**Free text box**

4. What else do you think could be done to encourage attendance in young adults?

**Free text box**

5. What else do you think could be done to improve delivery of DRS?

**Free text box**

Thank you for taking the time to complete this survey.

Table S1. Mean Score per TDF domain

Domain	Mean	SD
Social influences	3.58	1.00
Environmental context and resources	3.00	1.36
Social/professional role and identity	2.84	1.42
Knowledge	2.74	1.37
Beliefs about capabilities	2.69	1.12
Intention	2.47	1.07
Memory, attention and decision processes	2.31	0.95
Reinforcement	2.24	1.04
Skills	2.12	1.10
Behavioural regulation	2.12	1.03
Goals	1.97	0.99
Emotion	1.67	0.76
Optimism	1.54	0.71
Beliefs about consequences	1.08	0.30

The mean scores correspond to the extent to which participants agreed with each statement using a 5-point Likert scale (strongly agree=1; somewhat agree=2; neither agree or disagree=3; somewhat disagree=4; strongly disagree=5).

Mapping TDF Domains to BCTs

TDF domains identified from the BARS survey corresponding to barriers and enablers reported by HCPs to delivering DRS were mapped to behaviour change techniques (BCTs) and candidate intervention strategies to improve DRS uptake using a stepwise process.

This process yielded a set of candidate BCTs that could be operationalized to inform future interventions to improve DRS uptake in in young adults. The research team, working with a stakeholder advisory group consisting of diabetologists, ophthalmologists, screener/graders, young adults with diabetes, policy and diabetes charity representatives, provided examples of how the intervention might be delivered. Table S2 presents the links between TDF domains and theoretically coherent BCTs. Table 6 presents the links between the TDF domains identified from the BARS survey and theoretically coherent BCTs identified by the Theory and Techniques Tool. [1]

Table S2. Mapping barriers to potentially effective behaviour change techniques

Identified barrier	Corresponding TDF domain	Intervention function (Behaviour Change Wheel)	Behaviour Change Techniques	Intervention Target	Proposed operationalisation of selected intervention functions and BCTs
**Lack of confidence in discussing DRS with YA**	Beliefs about capabilities	Training<br><br>Education	*Instruction on how to perform the behaviour<br><br>Demonstration of the behaviour<br><br>Behavioural practice/rehearsal<br><br>Information about emotional consequences	HCP	Training could include;<br>• Actions HCPs can take to support, encourage and enable young adults to attend DES e.g., how to raise the issue of DES and check screening attendance in a non-judgmental way, how to facilitate referrals and access to convenient DES services, how to provide reassurance and address concerns around DR, complications/sight loss, and DES, reinforcing the benefits of screening [BCT: Instruction on how to perform the behaviour]<br>• Promotion of 'Language matter Diabetes' document which provides practical examples of language that will encourage positive interactions with people living with diabetes https://www.languagemattersdiabetes.com/ [BCT: Demonstration of the behaviour]

					• Videos demonstrating a HCP speaking to a YA with diabetes and practice/role playing different communication styles. [BCT: demonstration of the behaviour and behavioural practice/ rehearsal]<br>• A testimonial from a YA describing how negative communication impacted them [BCT: information about emotional consequences]
**Inflexible DRS booking systems and transient nature of young adults, who might be frequently moving between accommodation due to studies or employment**	Environmental context and resources	Service provision	Environmental restructuring	DESP	DESP service provision change could include:<br>• Increase availability of DES appointments for improved flexibility and choice in scheduling. Include the provision of evening/weekend appointments<br>• Allow self-booking of appointments and the choice to set the date of next appointment at end of current appointment<br>• Develop an easy system for YAs to inform the DESP of any change in personal details, e.g., address or change in GP. [BCT: Environmental restructuring]
**DESP staff should play more of a role in discussing screening results and diabetic self-management with YA**	Social professional role/ identity)	Training<br>Enablement	*Biofeedback<br><br>*Instruction on how to perform the behaviour<br><br>Demonstration of the behaviour<br><br>Social support<br><br>Framing/reframing<br><br>*Information about health consequences	YAs<br>HCP	Screeners who are suitably qualified providing initial indication of the likely result at DRS appointment, so YAs do not have to wait 2-3 weeks for results letter [BCT Biofeedback]. This may involve providing further training for screeners, and sample scripts/videos of how to discuss the results in an appropriate way [BCTs: Instruction on how to perform the behaviour; Demonstration of the behaviour]<br><br>Restructuring the content of the results letters so that the test result is accompanied by a clear explanation of the results (in lay language), reassurance about treatment options (phrased/framed positively) [framing/reframing; information about health consequences], and contact details for people YAs can discuss the results with (e.g. consultant/GP/someone from the DESP)

**Lack of integration and poor coordination between the DESP and other aspects of diabetes care**	Environmental Context and Resources	Enablement<br><br>Service provision	Environmental Restructuring<br><br>*Feedback on behaviour<br><br>Social comparison	Healthcare System	Evidence that increasedcommunication and multi-disciplinary teamwork leads to better outcomes. Therefore integrating eye screening with other diabetes services (e.g. ‘one-stop shop’ clinics) could potentially improve communication and outcomes. YA less likely to forget appointments and easier to arrange and monitor attendance and progress. [BCT: environmental restructuring]<br><br>Improving integration by facilitating communication between provider teams e.g. providing GPs with feedback and data on DRS attendance in local area [feedback on behaviour], benchmarked against similar GP practices [social comparison]. Highlighting the low attendance rates in YAs to persuade GPs that this is an issue/ draw their attention to it.

DR = diabetic retinopathy; DRS = diabetic retinopathy screening; YAs = young adults with diabetes; HCPs = healthcare professionals DESP= diabetic eye screening programme

*=BCTs used in published trials of interventions directed at HCPs to improve DRS uptake in a general population of people with diabetes. [2}

References

1. Human Behaviour Project. The Theory and Techniques Tool. Available at https://theoryandtechniquetool.humanbehaviourchange.org/tool (accessed 27.8.21)

2. Lawrenson, JG. Graham-Rowe E, Lorencatto F, Burr J, Bunce C, Francis JJ, Aluko P, Rice S, Vale L, Peto T. et al. Interventions to increase attendance for diabetic retinopathy screening. Cochrane Database Syst Rev 2018, 1, CD012054
